# The influence of muscle performance and fatigue on prognosis in patients with compensated liver disease

**DOI:** 10.1186/s12876-023-02885-2

**Published:** 2023-09-06

**Authors:** Ulrika Ekerfors, Magnus Simrén, Hanns-Ulrich Marschall, Daghan Demir, Axel Josefsson

**Affiliations:** 1 Department of Molecular and Clinical Medicine, Sahlgrenska Academy, University of Gothenburg, Göteborg, Sweden; 2 Institute of Internal Medicine Sahlgrenska Academy, University of Gothenburg, Sahlgrenska University Hospital, Göteborg, 41345 Sweden; 3 Center for Functional Gastrointestinal and Motility Disorders, University of North Carolina at Chapel Hill, Chapel Hill, NC USA

**Keywords:** Liver disease, Sarcopenia, Liver transplantation, Fatigue

## Abstract

**Background:**

Poor muscle function is associated with a negative prognosis in advanced liver disease but the impact in compensated chronic liver disease is unknown. Similar prognostic uncertainty applies to fatigue. We aimed to assess the prognostic value of muscle performance and fatigue in a cohort of patients with compensated chronic liver disease.

**Methods:**

We followed 241 patients with compensated chronic liver disease included in a study between 2010 and 2014. Subjects were 52 ± 15 years (mean ± SD; 134 females). All subjects performed four muscle function tests: “Timed Up and Go” test, walking speed, handgrip strength, and standing heel-rises. Fatigue was evaluated by fatigue impact scale. Follow up data was acquired through hospital records and registries.

**Results:**

During follow up of 6.75 ± 1.4 years, 13 patients died (5.5%) and 11 (4.5%) patients underwent liver transplantation. A timed up and go over 10 s was not significantly associated with a lower survival (Kaplan-Meier, log rank test p = 0.132), or with transplant free survival (p = 0.543), Fig. 3. It was also not specifically associated with liver related causes of death (p = 0.597). The other physical functioning tests and fatigue were not significantly associated with mortality or transplant-free survival (p > 0.05 for all) except for maximal walking speed (2.2 vs. 1.9 m/s, p = 0.007).

**Conclusions:**

Our study suggests that muscle function and fatigue are not key prognostic factors in compensated chronic liver disease. However, further confirmation in future studies is needed.

## Introduction

Poor muscle function is a frequent finding in patients with advanced chronic liver disease (CLD). A common cause of muscle dysfunction in this population is sarcopenia, which has previously been shown to be a negative prognostic factor [1]. The prevalence of sarcopenia in patients with advanced CLD has been estimated to be as much as 37.5% [2]. However studies regarding the prevalence of sarcopenia and poor muscle function in non-advanced CLD are few [3], and muscle function tests such as the Timed up and go test have been linked to a worse prognosis in several other populations [4, 5]. The suggested definitions of sarcopenia have moved to focus on muscle function rather than solely on muscle mass [6], which has not been studied to the same extent in compensated CLD, but the etiology of sarcopenia and poor muscle performance is likely multifactorial in most patients with liver disease and possibly different in some aspects in advanced versus non-advanced CLD [7, 8]. Previous studies have also shown that sarcopenia in patients with decompensated liver disease is related to hepatic encephalopathy, mortality, worse outcome following liver transplantation, and other complications of decompensated chronic liver disease [2, 7]. However, the impact of muscle function on prognosis in compensated CLD is unknown.

Another common and well-known symptom in patients with CLD is fatigue which has also been linked to poor muscle function [3]. Peripheral muscle dysfunction has been suggested as a contributing factor to fatigue, at least in Primary Biliary Cholangitis (PBC) [9–11]. However, the impact of fatigue on prognosis in compensated CLD is unknown.

## Methods

Our primary aim was to assess the potential prognostic value of muscle performance and fatigue in a cohort of patients with a variety of common CLDs on mortality and transplant-free survival. Our secondary aim was to assess the impact of muscle function, physical activity, and fatigue on the incidence of liver decompensation.

**Fig. 1:**
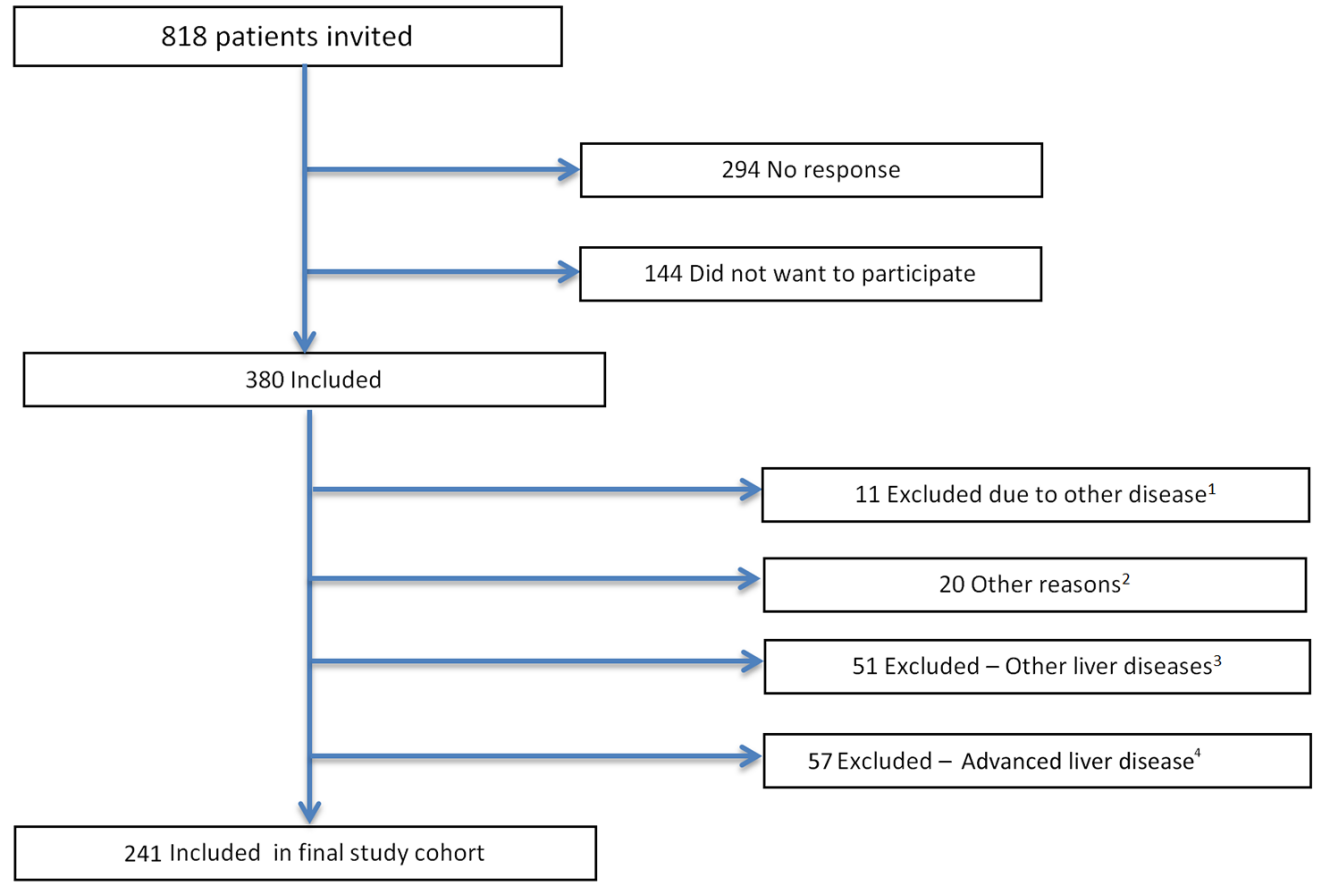
Subjects included in the study cohort. (1) Malignancy, stroke, severe hypertension, severe hypothyroidism, mobility impairment. (2) Patient participated in another drug study, 1 started interferon treatment, 6 dropouts, 12 excluded due to language difficulties. (3) 9 with cryptogenic cirrhosis, 21 alcoholic cirrhosis, 21 Non alcoholic fatty liver disease. (4) Subjects with previous decompensated liver disease

We followed a prospectively consecutively recruited cohort of patients with compensated CLD (Child-Pugh B/C were excluded) that were enrolled in a study on muscle function and CLD in our institution (Sahlgrenska University Hospital in Gothenburg, Sweden) between 2010 and 2014 [3]. All subjects with compensated CLD caused by autoimmune hepatitis (AIH), PBC, primary sclerosing cholangitis (PSC), chronic hepatitis B (HBV) and chronic hepatitis C (HCV) were invited to participate in a study on muscle function and fatigue. Subjects with any sign of deteriorated liver function as evidenced by a Child-Pugh (CP) score > 6 were excluded, to minimize metabolic or drug treatment confounders. Overt hepatic encephalopathy was clinically excluded by an experienced hepatologist. We also excluded all patients with other causes of CLD than PBC, PSC, AIH, HBV or HCV, in particular alcoholic liver disease, but also NAFLD and cryptogenic liver cirrhosis [12]. Subjects with impaired mobility or other severe medical conditions including, but not limited to, neurologic, cardiovascular, or malignant conditions were also excluded which could be considered as a major contributor to impaired muscle function tests and fatigue (Fig. 1).

The study protocol conforms to the ethical guidelines of the 1975 Declaration of Helsinki (6th revision, 2008) and was approved by the Regional Ethical Review Board in Gothenburg (Dnr 401 − 10, 2010-10-15). All subjects included gave written consent to participate in the study.

### Follow-up

Patients were followed from inclusion in the original study [3], until death, liver transplantation or last day of available follow-up data. Three strategies were used: (1) Date and cause of death were obtained from the National Cause of Death Register; (2) Scrutinization of local, regional, and national medical records (digital archive available in Sweden) for information on any liver related complications such as decompensation (ascites, encephalopathy, bleeding esophageal varices) or development of cirrhosis such as radiologic or clinical signs; (3) Information if the patient underwent liver transplantation during follow up was registered at the transplantation unit. Linkage to the registries was possible through the unique personal number assigned to all Swedish residents.

### Decompensation

Decompensated liver disease during follow up was defined as new onset ascites, overt hepatic encephalopathy, or bleeding esophageal varices [13].

### Measurement of muscle strength

Four standardized tests on muscle function were performed by all subjects. A healthy reference population was used to adjust the results for age and sex [14, 15].

### Walking test

The time it takes for subjects to walk 30 m at their normal walking speed on a flat ground is measured and then again when the same distance is passed at their maximum speed [14].

### Hand grip strength

By using Grippit®, an electronic grip force instrument, (AB Detector, Göteborg, Sweden) [16] we registered the peak maximum grip force and the mean grip force value of a 10-second period with sustained grip (the right hand only) in seated position and measured in Newtons.

### The standing heel-rise test (SHT)

Patients performed a maximal heel rise test on a 10-degree tilted wedge (right foot only) in a predetermined pace every other second using a metronome. Subjects were allowed to touch the wall for balance. Failure to keep the pace or when the subjects were too exhausted to complete a maximal heel rise. The number of heel-rises was counted [17].

### Timed “Up & Go” test

The time (seconds) it takes to stand up from a standard arm chair, walk a distance of 3 m, turn back and walk back (total 6 m) to the chair and sit down again is measured [18]. Previous studies have used different cut offs to define normal performance of the test. We tested a 10 s cut off as previously suggested [4, 5].

### Questionnaires

All patients completed two validated questionnaires to assess the level of physical activity and the level of fatigue:

### Saltin-Grimby Physical Activity Level Scale

The participation in recreational and work-related activities are graded according to a seven-grade scale from 0 (no regular physical activity) to 6 (regular strenuous physical activity several times/week), and subjects were grouped into high (level 5–6) vs. low physical activity (0–4) [19].

### Fatigue impact scale (FIS)

This questionnaire uses questions on cognitive, physical, and psychosocial fatigue. A higher score corresponds to higher fatigue (0-160) and a cut off level of 40 for high-degree fatigue has previously been used [20–24].

### Statistics

Data are presented as mean and standard deviation (SD) or as median and range and as number (n) and percentages, as appropriate. Two groups were compared by using the chi square test for categorical variables. Correlations between continuous variables were analyzed by the Spearman rank order correlation test (rho), whereas the Mann-Whitney U test or the Student T-test were used for comparisons of continuous variables between two groups, respectively depending on normal distribution or not. We used Kaplan Meier analyses to test the outcome variables mortality and transplant free survival. We also corrected survival analyses for variables associated with a lower survival in Kaplan-Meier analyses using a Cox regression model by entering variables chosen *a priori*, age and sex. Multicollinearity was assessed with VIF’s (variance inflation factors), which were low (< 3).

We tested the variables described in the methods section on physical function and fatigue and the potential relationship to the outcome variables. In order to consider a muscle function test as abnormal, a comparison with a healthy reference population from a previous study at our center was performed, stratified in age by decades and by sex [14, 15], and tests that were lower than the normal values, i.e. below 100% were classified as abnormal. All tests were two-tailed and significance was accepted at the 5% level. Statistics were calculated with SPSS v 21.0.

## Results

### Subjects

In all, 270 CLD outpatients were included in the original study cohort, however we excluded 29 subjects with a history of hepatic decompensation. Diagnoses in the remaining 241 subjects (134 females) were PBC; n = 41, PSC; n = 42, AIH; n = 48, and viral hepatitis HBV, n = 51; HCV, n = 59, mean age at inclusion was 51.5 (SD 14.5) years, Table 1 for details.

**Table 1:** Characteristics of subjects included in the study cohort at baseline and at last follow up

	Baseline	Last Follow up
**Sex**	134 female (55%), 107 male	126 female (55%), 101 male
**Age**	51.5 (14.5) years	58.5 (14) years
**Body Mass Index**	25 (4.5) kg/m^2^	26.5 (6) kg/m^2^
**Creatinine** **Hemoglobin** **Bilirubin** **Albumin** **International normalized ratio** **Thrombocyte count**	75 (17) µmol/L 140 (14) g/L 9.5 (5.5) µmol/L 40 (4) g/L 1.0 (0.1) 257 (80) *10^9^	78 (28) µmol/L 137 (17) g/L 13 (36) µmol/L 40 (5) g/L 1.0 (0) 233 (78) *10^9^
**Tobacco smoker**	50 (20%)	

Data presented as mean (standard deviation) and n (%) as appropriate

### Follow up

The subjects were followed for a mean of 6.75 (SD 1.4) years, mean age at follow up was 58.5 years (SD 14). During this time, 13 patients died (5.5%) and 11 (4.5%) patients underwent liver transplantation. Lab tests and body composition at inclusion and at last follow up were similar (Table 1).

### Muscle function and fatigue

**Fig. 2:**
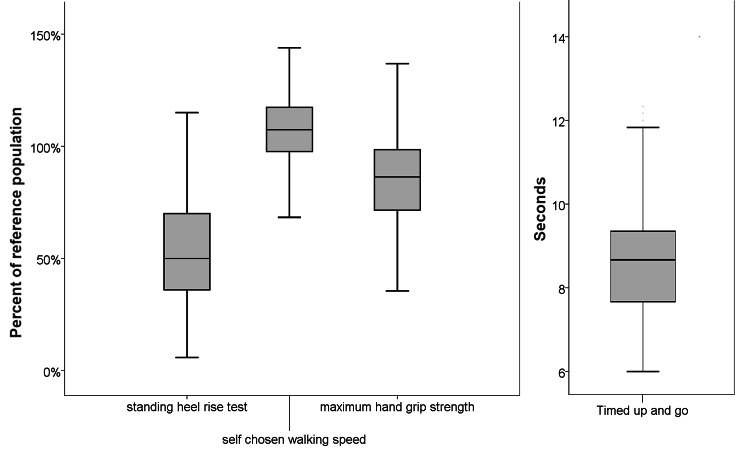
The distribution of muscle function tests. Standing heel rise test, self chosen walking speed, and maximum grip strength is shown as compared with a normal population. The results from the timed up and go test is shown in seconds

The mean standing heel rises was 13 (SD 6.5), mean self-chosen walking speed was 1.5 m/s (SD 0.25), mean maximal walking speed was 2.1 m/s (SD 0.4), maximal grip strength 331 N (SD 129), and mean time to complete the timed up and go test was 8.7 s (SD 1.5). A total of 41 subjects had a timed up and go of 10 s or more. At baseline muscle function was impaired in several tests compared with a healthy control population (Table 2). The distribution of muscle function tests compared with a normal population is shown in Fig. 2. The results from the FIS are presented in Table 2. Ninety-five subjects (39.5%) had a FIS score above 40.

**Table 2:** Results from Fatigue impact scale and proportion of subjects with a muscle function test value below 100% of healthy subjects along side with mean and standard deviation.

Physical function tests	
Standing heel rise, below reference	13 (SD 6.5), 218/241 (90.5%)
Maximal hand grip strength, below reference	331 N (SD 129), 187/241 (77.5%)
Walking speed (self chosen), below reference	1.5 m/s (SD 0.25), 73/241 (30%)
Walking speed (maximal), below reference	2.1 m/s (SD 0.4), 113/241 (47%)
Timed up and go, above 10 seconds	8.7 s (SD 1.5), 41/241 (17%)
Physical activity level (0–6), high level (5–6)	3.3 (1.3), 36/241 (15%)
**Fatigue impact scale score**	
Total	38.5 (36.5)
Physical	10 (10)
Cognitive	10 (9)
Psycho-social	18.5 (17.5)

Data presented as mean (standard deviation) and n (%) as appropriate

We also analysed the potential association between all muscle function tests and Child Pugh score, MELD score, PK (INR), Bilirubin, and albumin. However, no muscle test was significantly correlated with liver function tests (p > 0.05 for all).

### Outcome

#### Mortality and liver transplantation

**Fig. 3:**
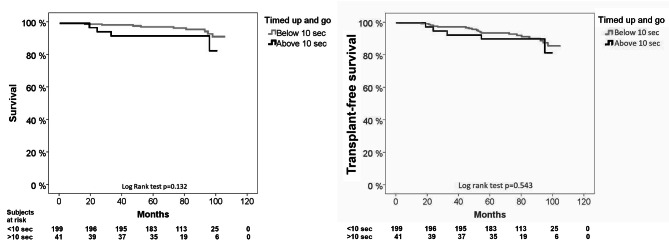
Kaplan-Meier test; subjects with a Timed up and go of 10 s or more, p value represents Log rank test

During the follow up period, 13 patients died (5.5%) and 11 (4.5%) patients underwent liver transplantation. Four deaths were liver related, 2 cardiovascular, 2 malignancies that were not liver related, 3 due to severe infection, and 2 because of other causes. A timed up and go over 10 s was not significantly associated with a lower survival (Kaplan-Meier, log rank test p = 0.132), or with transplant free survival (p = 0.543), Fig. 3. It was also not specifically associated with liver related causes of death (p = 0.597).

We also did a Cox regression to correct for age and sex with the timed up and go test, but it was not significant for survival or transplant free survival, when correcting for age and sex. The other physical functioning tests were not significantly related with survival; standing heel rises (survivors 13.5 vs. non survivors 11, p = 0.251), average handgrip strength (298 vs. 250 N, p = 0.153), maximal hand grip strength (335 vs. 280 N, p = 0.129), self-chosen walking speed (1.50 vs. 1.37 m/s, p = 0.065), except for maximal walking speed (2.2 vs. 1.9 m/s, p = 0.007). When dichotomizing the cohort according to the normal values of the control population, no physical function test besides timed up and go was significantly associated with mortality or transplant free survival (Kaplan-Meier, log rank test p > 0.05 for all).

Fatigue was not a significant predictor of outcome. Using the FIS score of 40 as the cut-off for fatigue, we could not demonstrate any association between survival or transplant-free survival and FIS score (Kaplan-Meier, log rank test p = 0.096 for survival and p = 0.071 for transplant-free survival respectively). We also analysed the subdomains of the FIS score, however none were significantly related to mortality, FIS physical (survivors 10.0 vs. non-survivors 7.2, p = 0.336), FIS cognitive (10.3 vs. 5.1, p = 0.057), and FIS psychosocial (19.0 vs. 9.9, p = 0.073), or transplant-free survival, FIS physical (9.9 vs. 8.7, p = 0.561), FIS cognitive (10.3 vs. 7.5, p = 0.170), and FIS psychosocial (19.1 vs. 13.4, p = 0.139).

### Decompensation

Decompensated liver disease during follow-up occurred in 33/241 subjects (13.5%). It was related to self-chosen walking speed below reference (12/73 (16.5%) vs. 11/168 (6.5%), p = 0.016). However, it was not related to age, sex, fatigue, physical activity, or other physical functioning tests.

## Discussion

In this study we followed a cohort of subjects with compensated chronic liver disease for several years and assessed the potential prognostic implication of physical function and fatigue. The key findings were that muscle function tests at baseline were essentially not correlated with prognosis at follow up.

Physical function in our cohort was generally lower compared with a control population, which suggest that several subjects in our cohort suffer from or are at risk of developing sarcopenia. This is a finding that has been shown in studies in advanced CLD or cirrhosis [7]. We did not use advanced body composition metrics such as Dual-energy X-ray absorptiometry, which would have added information to our assessment of sarcopenia [6]. However we did use several well validated muscle function tests to detect poor muscle function, which is now considered more important than muscle mass per se as it displays a stronger link with outcome in other populations [6]. Several previous studies have also shown that sarcopenia is an important predictor of survival in subjects with cirrhosis [2]. This is in contrast with our results, where we could not demonstrate a clear correlation between most muscle function tests and survival. This is likely explained by the fact that all subjects in our cohort were compensated at inclusion. Thus, the effects of sarcopenia in a decompensated liver disease population is likely not generalizable to our cohort of compensated liver disease. However, muscle function and muscle mass does play an important role in liver disease, but the exact timing for when it starts to impact survival is still yet to be determined. The difference in survival between subjects with poor muscle function and normal muscle function in our cohort was not statistically significant except for walking speed. However, we cannot make any conclusions of cause and effect from our data, but other studies have shown that frail individuals without liver disease where timed up and go has been shown to predict premature mortality [5, 25]. Previous studies have also shown that a long timed up and go is a risk factor for falls in subjects with liver cirrhosis [25, 26]. The time to complete timed up and go has also been shown to decrease if the subject engages in an exercise program, which suggests that physical training, even in subjects with compensated CLD could potentially be beneficial [26].

The optimal cut-off to use for the timed up and go test in our population is not known. We used the 10 s cut off for the timed up and go test. But there are also studies suggesting a long cut off, for example a 14 s cut-off, but this population only comprised very few individuals, so the results would have been difficult to interpret and was not included in the study. There are also studies suggesting an even longer time as cut-off for the timed up and go test [27]. However, we did not perform further analyses on longer cut-off times as our cohort did not contain any subjects with an even slower timed up and go test. Based on the results in this study, we cannot exclude that timed up could have an association with with prognosis. Even though the other muscle functions tests did not display a statistically significant association with outcome, all tests showed the same pattern, i.e. that survivors compared with the others tended to have a better muscle function across all tests. Thus, a type 2 error cannot be excluded.

Fatigue was not associated with a lower overall survival or transplant free survival in our cohort. A previous study has suggested that fatigue in primary biliary cholangitis was associated with a lower rate of survival at a 4 year follow up [23]. The reason for this finding is unknown, but we could not confirm this in our study. The cohorts are not entirely comparable regarding liver disease severity and in our cohort a broader range of liver diseases were also included, which could explain that we could not demonstrate the same findings. However, the association between fatigue and severity of liver disease is low [28, 29].

Our study has several strengths. We had a relatively long follow-up period. However, ideally this should have been even longer to record a larger number of outcome events, especially when studying a group with patients with compensated liver disease. Previous studies suggest that significant mortality and decompensation in chronic liver disease and compensated cirrhosis generally occurs at periods of longer follow-up than the period used in our cohort [30]. This would support the hypothesis that a longer follow-up period would have been required in our study. Furthermore, we had a relatively large cohort in which muscle function was characterized in a structured manner. We also used validated registries, which have a high degree of reliability, and we also had access to the Swedish electronic national medical records, which were scrutinized [31]. During the year 2020, only 0.9% of subjects who died in Sweden did not receive a certificate of cause of death [32]. Another strength in our study is the variety of muscle function tests that all subjects underwent at inclusion done by a single investigator, ensuring a standardized procedure. By using validated tests for both upper and lower body strength, and functional test such as the timed up and go, we achieved a comprehensive assessment of the muscle function. We also used validated questionnaires for both fatigue and the level of physical activity. The FIS has also been used in liver populations in previous studies [33, 34].

Our study also has some limitations. We did not have any formal follow-up appointment to record deterioration of liver disease, muscle function, or assessment using questionnaires. Thus, we cannot accurately determine in which way the liver function deteriorated and the change in muscle function or fatigue during the follow-up period. As we had a long follow up period, there are also some limitations with this, as many confounding events may occur during this follow up time, and we could not account for all which could have impacted our results. It is also possible that the muscle function and mass changed during follow up, which could influence the results at follow up. Furthermore, even though we had a solid strategy for follow-up, certain data may be missing. For example, subjects moving abroad are not registered in same way as subjects remaining within Sweden. Liver transplantations in Sweden are only done at two specialized centres, with one being our centre. So, we do not expect any missing data for liver transplantation, provided that the subjects did not move abroad. Another limitation is that only a few subjects underwent liver transplantation or died, which could have impacted our results and type 2 errors cannot be ruled out. Additionally, we included a selected population of liver diseases; autoimmune, cholestatic, and viral liver disorders, thus we cannot generalize our findings to other liver disorders. Finally, our follow-up period stopped a few months prior to the start of the SARS-CoV-2 pandemic, so we cannot assess the impact this would have had on our population.

In conclusion, assessment of muscle function could potentially serve as a prognostic indicator in patients with compensated chronic liver disease. The prognostic value of muscle function in patients with compensated liver disease should be evaluated further in future trials. It would also be of value to assess the potential impact of physical training to improve the clinical outcome among subjects with compensated chronic liver disease.

## Data Availability

The datasets generated and/or analysed during the current study are not publicly available as this was not stated in the ethical approval but could be available from the corresponding author on reasonable request and renewed ethical board application.

## Abbreviations

CLD: Chronic liver disease
PBC: Primary Biliary Cholangitis
AIH: Autoimmune hepatitis
PSC: primary sclerosing cholangitis
HBV: chronic hepatitis B
HCV: chronic hepatitis C
CP: Child-Pugh
SHT: Standing heel-rise test
FIS: Fatigue Impact Scale
SD: Standard Deviation
N: Newton

## References

[1] Hanai T, Shiraki M, Nishimura K (2015). Sarcopenia impairs prognosis of patients with liver cirrhosis. Nutrition.

[2] Tantai X, Liu Y, Yeo YH et al. *Effect of sarcopenia on survival in patients with cirrhosis: a meta-analysis*. J Hepatol, 2021. 10.1016/j.jhep.2021.11.006 34785325

[3] Ekerfors U, Sunnerhagen KS, Westin J (2019). Muscle performance and fatigue in compensated chronic liver disease. Scand J Gastroenterol.

[4] Chun S, Shin DW, Han K (2021). The timed up and go test and the ageing heart: findings from a national health screening of 1,084,875 community-dwelling older adults. Eur J Prev Cardiol.

[5] Son KY, Shin DW, Lee JE (2020). Association of timed up and go test outcomes with future incidence of cardiovascular disease and mortality in adults aged 66 years: korean national representative longitudinal study over 5.7 years. BMC Geriatr.

[6] Cruz-Jentoft AJ, Bahat G, Bauer J (2019). Sarcopenia: revised european consensus on definition and diagnosis. Age Ageing.

[7] Tandon P, Montano-Loza AJ, Lai JC (2021). Sarcopenia and frailty in decompensated cirrhosis. J Hepatol.

[8] Sung MJ, Lim TS, Jeon MY (2020). Sarcopenia is independently Associated with the degree of liver fibrosis in patients with type 2 diabetes Mellitus. Gut Liver.

[9] Hollingsworth KG, Newton JL, Robinson L (2010). Loss of capacity to recover from acidosis in repeat exercise is strongly associated with fatigue in primary biliary cirrhosis. J Hepatol.

[10] Les I, Doval E, Flavia M (2010). Quality of life in cirrhosis is related to potentially treatable factors. Eur J Gastroenterol Hepatol.

[11] Goldblatt J, James OF, Jones DE (2001). Grip strength and subjective fatigue in patients with primary biliary cirrhosis. JAMA.

[12] Pugh RN, Murray-Lyon IM, Dawson JL (1973). Transection of the oesophagus for bleeding oesophageal varices. Br J Surg.

[13] de Franchis R, Bosch J, Garcia-Tsao G (2022). Baveno VII - renewing consensus in portal hypertension. J Hepatol.

[14] Brodin E, Ljungman S, Hedberg M (2001). Physical activity, muscle performance and quality of life in patients treated with chronic peritoneal dialysis. Scand J Urol Nephrol.

[15] Brodin E, Ljungman S, Sunnerhagen KS (2008). Rising from a chair: a simple screening test for physical function in predialysis patients. Scand J Urol Nephrol.

[16] Nordenskiold U (1990). Elastic wrist orthoses. Reduction of pain and increase in grip force for women with rheumatoid arthritis. Arthritis Care Res.

[17] Svantesson U, Osterberg U, Thomee R (1998). Muscle fatigue in a standing heel-rise test. Scand J Rehabil Med.

[18] Podsiadlo D, Richardson S (1991). The timed “Up & Go”: a test of basic functional mobility for frail elderly persons. J Am Geriatr Soc.

[19] Grimby G, Frandin K. *On the use of a six-level scale for physical activity*. Scand J Med Sci Sports, 2017. 10.1111/sms.12991 29027263

[20] Fisk JD, Ritvo PG, Ross L (1994). Measuring the functional impact of fatigue: initial validation of the fatigue impact scale. Clin Infect Dis.

[21] Prince MI, James OFW, Holland NP (2000). Validation of a fatigue impact score in primary biliary cirrhosis: towards a standard for clinical and trial use. J Hepatol.

[22] Jones DE, Gray JC, Newton J (2009). Perceived fatigue is comparable between different disease groups. QJM.

[23] Jones DE, Bhala N, Burt J (2006). Four year follow up of fatigue in a geographically defined primary biliary cirrhosis patient cohort. Gut.

[24] Goldblatt J, Taylor PJ, Lipman T (2002). The true impact of fatigue in primary biliary cirrhosis: a population study. Gastroenterology.

[25] Nardelli S, Gioia S, Ridola L, et al. Risk of falls in patients with cirrhosis evaluated by timed up and go test: does muscle or brain matter more? Dig Liver Dis; 2021. 10.1016/j.dld.2021.06.019 34233863

[26] Roman E, Garcia-Galceran C, Torrades T (2016). Effects of an Exercise Programme on Functional Capacity, Body Composition and Risk of Falls in patients with cirrhosis: a Randomized Clinical Trial. PLoS ONE.

[27] Beauchet O, Fantino B, Allali G (2011). Timed up and go test and risk of falls in older adults: a systematic review. J Nutr Health Aging.

[28] Cauch-Dudek K, Abbey S, Stewart DE (1998). Fatigue in primary biliary cirrhosis. Gut.

[29] Bjornsson E, Simren M, Olsson R (2004). Fatigue in patients with primary sclerosing cholangitis. Scand J Gastroenterol.

[30] D’Amico G, Morabito A, D’Amico M (2018). Clinical states of cirrhosis and competing risks. J Hepatol.

[31] SWEDEN OSO. *Statistics – Health and Medical Care, Inpatient diseases in Sweden 1987–2008* ISBN 978-91-86301-52-1.

[32] Socialstyrelsen. *Declaration of quality - Official statistics of mortality 2020 in Sweden* 2021.

[33] Swain MG, Jones DEJ. Fatigue in chronic liver disease: new insights and therapeutic approaches. Liver Int; 2018. 10.1111/liv.13919 29935104

[34] Kalaitzakis E, Josefsson A, Castedal M et al. *Factors related to fatigue in patients with cirrhosis before and after liver transplantation* Clin Gastroenterol Hepatol, 2012. 10(2): p. 174 – 81, 181 e1. 10.1016/j.cgh.2011.07.029 21839709

